# Neonate Intestinal Immune Response to CpG Oligodeoxynucleotide Stimulation

**DOI:** 10.1371/journal.pone.0008291

**Published:** 2009-12-14

**Authors:** Sonia Lacroix-Lamandé, Nicolas Rochereau, Roselyne Mancassola, Mathieu Barrier, Amandine Clauzon, Fabrice Laurent

**Affiliations:** Laboratoire Contrôle et Immunologie des Maladies Entériques du Nouveau-né, UR1282 Infectiologie Animale et Santé Publique, INRA de Tours, Nouzilly, France; Institut Pasteur, France

## Abstract

**Background:**

The development of mucosal vaccines is crucial to efficiently control infectious agents for which mucosae are the primary site of entry. Major drawbacks of these protective strategies are the lack of effective mucosal adjuvant. Synthetic oligodeoxynucleotides that contain several unmethylated cytosine-guanine dinucleotide (CpG-ODN) motifs are now recognized as promising adjuvants displaying mucosal adjuvant activity through direct activation of TLR9-expressing cells. However, little is known about the efficacy of these molecules in stimulating the intestinal immune system in neonates.

**Methodology/Principal Findings:**

First, newborn mice received CpG-ODN orally, and the intestinal cytokine and chemokine response was measured. We observed that oral administration of CpG-ODN induces CXC and CC chemokine responses and a cellular infiltration in the intestine of neonates as detected by immunohistochemistry. We next compared the efficiency of the oral route to intraperitoneal administration in stimulating the intestinal immune responses of both adults and neonates. Neonates were more responsive to TLR9-stimulation than adults whatever the CpG-ODN administration route. Their intestinal epithelial cells (IECs) indirectly responded to TLR9 stimulation and contributed to the CXC chemokine response, whereas other TLR9-bearing cells of the lamina-propria produced CC chemokines and Th1-type cytokines. Moreover, we showed that the intestine of adult exhibited a significantly higher level of IL10 at homeostasis than neonates, which might be responsible for the unresponsiveness to TLR9-stimulation, as confirmed by our findings in IL10-deficient mice.

**Conclusions/Significance:**

This is the first report that deciphers the role played by CpG-ODN in the intestine of neonates. This work clearly demonstrates that an intraperitoneal administration of CpG-ODN is more efficient in neonates than in adults to stimulate an intestinal chemokine response due to their lower IL-10 intestinal level. In addition we report the efficiency of the oral route at inducing intestinal chemokine responses in neonate that might be taken into consideration for further vaccine development against neonatal diseases.

## Introduction

Neonates and infants have an increased susceptibility to infection due to inherent limitations of their immune system. It is believed that neonates exhibit functionally impaired antigen presentation, shorter lived and weaker antibody responses, a Th2-type immune response bias and a decreased overall cell-mediated immune response if compared with adults [1]. However, under appropriate conditions, neonates can develop immune responses to vaccination that are qualitatively and quantitatively similar to adults [2]–[5].

The mucosal surfaces are the primary site of entry of most pathogens and thus the development of vaccines delivered mucosally should help to promote mucosal immune responses, as well as providing a safe, readily acceptable method for inducing systemic immune responses. One of the major drawbacks in the development of mucosal vaccines has been the lack of effective mucosal adjuvants [6].

Bacterial DNA has direct immunostimulatory effects due to the presence of unmethylated cytosine–guanine dinucleotides (CpG) within a particular base context (CpG motifs) [7]. Synthetic oligodeoxynucleotides (ODN) that contain one or more CpG motifs and mimic bacterial DNA are now recognized as promising adjuvants. CpG-ODN has strong adjuvant activity due to its many effects on both innate and adaptive immune responses. Indeed, CpG ODN can directly activate monocytes, macrophages and dendritic cells to secrete IFN-α/β, IL-6, IL-12, GM-CSF, chemokines, and TNF-α, which in turn stimulate T-cells and natural killer (NK) cells to produce additional cytokines, such as IFN-γ. CpG-ODN also causes up-regulation of co-stimulatory molecules and MHC class II molecules, improving antigen presentation by professional cells. In response to CpG-ODN stimulation, B-cells proliferate, secrete immunoglobulins and provide a T-helper function by strong Type 1-like patterns of cytokine production that is dominated by IL-12 and IFN-α, with little secretion of Type 2 cytokines [8], [9].

The use of CpG-ODN as a potent Th1-like adjuvant for mucosal vaccinations with various antigens has been largely described for hepatitis B, measles virus, and HSV-1 [10]. Moreover, many preclinical and early clinical trials indicate that synthetic CpG-ODN has potent immunostimulatory effects and can enhance the activity of various anti-cancer treatments [11]. These immunostimulatory properties of CpG-ODN can also be efficiently used without antigens to control infectious diseases such as listeriosis [12] and *Helicobacter pylori* infection [13]. We have previously reported that CpG-ODN administered orally to newborn mice confers 90% protection against *Cryptosporidium parvum* enteric infection [14]. However, despite increasing knowledge about the immunostimulatory effects of CpG-ODN systemically administered in adult mice, there is still limited information on the efficiency of such molecules on the intestinal innate immune system when delivered to newborns.

We therefore studied the mucosal immune response following an oral administration of CpG-ODN to neonates, and investigated the contribution of intestinal epithelial cells (IECs) and the neonatal environment in this response.

## Materials and Methods

### Reagents

Rat anti-neutrophil clone 7/4, rat anti-B220 clone RA3-6B2, rat anti CD8α clone YTS105.18 and hamster anti-CD11c clone HL3 were purchased from Serotec, rat anti-CD4 clone YTS191.1.2 from Cedarlane and were used for immunofluorescence analysis. The expression of cell surface molecules by flow cytometry was analyzed using the following antibodies purchased from BD pharmingen: anti-CD11c antibodies conjugated with FITC or PE (HL3), anti-CD8α-PE (53-6.7), anti-CD4 (H129.19), and anti-CD19 (ID3) antibodies.

CpG-ODN 1668 (for murine cells) was purchased from Sigma-Aldrich.

### Mice and In Vivo Treatment

C57BL/6J female mice with or without litters were housed under specific pathogen-free conditions, and were maintained at constant temperature and humidity, with food and water given ad libitum. C57BL/6 IL10-deficient mice were kindly provided by Richard Lo-Man (Institut Pasteur, France) and C57BL/6 TLR9-deficient mice by Shizuo Akira (Osaka University, Japan). Eight-day-old neonates and 6-8-week-old adult mice received CpG-ODN by oral (20 µg/g) and by intraperitoneal (10 µg/g) route.

Experimental protocols were designed in compliance with recommendations of the French law (Décret: 2001-464 29/05/01) and EEC (86/609/CEE) for the care and use of laboratory animals.

### Purification of Intestinal Epithelial Cells (IEC)

Purification of IEC was performed as described previously by Perreault and Beaulieu with some modifications [15]. The small intestine was opened longitudinally, washed in phosphate-buffered saline (PBS) and cut into 5-mm fragments. The fragments obtained were transferred into a flask with ice-cold Matrisperse (BD biosciences), and incubated at 4°C for 8–10 h without agitation. The flask was then gently shaken to separate the epithelium. The mixture was passed through 100 µm and then 60 µm filters and epithelium was recovered on the 60 µm filter. After washes, epithelial cells were individually separated by trypsination. Cells were then stained with phycoerythrin-labeled anti-CD4, CD8, CD11b, and NK1.1 antibodies. Cells were sorted by MoFlo (Dako) according to SSC-FSC and PE non-positivity. The viability of purified IEC after sorting was about 85%. Two hours after in vitro cultivation, viability of IEC was about 60%. For neonates, ten intestines were pooled to obtain one value.

### Immunohistology

Pieces of ileum from neonatal mice were frozen at −80°C in embedding matrix for immunohistological studies. Frozen sections (7 µm) were cut and fixed in acetone gradient and incubated with primary antibodies for 2 h at room temperature. Sections were washed several times in PBS, and were then incubated with an anti-rat AlexaFluor 594 conjugate (Molecular Probes) and an anti-hamster biotin conjugate (Jackson ImmunoResearch), for 1 h at room temperature. Sections were finally washed and incubated with ExtrAvidine fluorescein isothiocyanate conjugate (Sigma) for 20 minutes. Sections were counterstained with Hoechst nuclear dye and observed on a Zeiss fluorescence microscope with the Axiovision software (Zeiss, Göttingen, Germany).

### Collection of Mesenteric Lymph Node Cells and FACS

Mesenteric lymph nodes (MLN) were removed from neonates and placed in Petri dishes with cold RPMI 1640 medium (Invitrogen). They were crushed on 60 µm Blutex and cells were spun for 5 min at 1,500 rpm at 4°C. Cells were resuspended in PBS supplemented with 1% FCS. We used 10^6^ cells for each staining and non-specific staining was prevented by first blocking the cells with anti-CD16/CD32 antibody (2.4G2; BD Pharmingen). Cells were incubated with the antibodies for 30 minutes, fixed in 2% paraformaldehyde and sorted on a Becton-Dickinson fluorescence-activated cell sorter (FACS) with the CellQuest-Pro software.

### mICcl2 and CMT93 Cultures

The murine cell line mICcl2 was kindly provided by A. Vandewalle [16]. This immortalized cell line is from the crypt of the small intestine of mice and was maintained at 37°C in the presence of 5% CO_2_ on growth medium containing Dulbecco modified Eagle medium and Ham's F12 medium at a ratio of 1∶1 (vol/vol), 60 nM sodium selenate, 5 µg of transferrin per ml, 2 mM glutamine, 5 µg of insulin per ml, 50 nM dexamethasone, 5 nM triiodothyronine, 10 ng of epidermal growth factor per ml, 20 mM D-glucose, 2% fetal calf serum, and 20 mM HEPES (pH 7.4). Murine rectal adenocarcinoma (CMT-93) cell lines were grown in DMEM supplemented with 10% heat-inactivated fetal calf serum, 4 mM glutamine, 100 U of penicillin per ml, 100 µg of streptomycin per ml, and 1% nonessential amino acids in a 5% CO_2_ incubator at 37°C. Cells were seeded into 6-well plastic tissue culture plates and were stimulated when they reached confluence.

### RNA Extraction and Reverse-Transcription (RT) Reaction

Ilea were removed from adult and neonate mice. Ilea were placed in TRIzol (InVitrogen) and were crushed with an Ultra-Turrax homogenizer. The samples were centrifuged at 8,000×*g* to eliminate debris, and the supernatants were stored at −80°C until further processing. RNA was extracted according to the manufacturer's instructions and quantified by measuring absorbance at 260 nm. RNA quality was estimated by agarose gel electrophoresis, using ethidium bromide staining.

### Analysis of Inflammatory Cytokines, Chemokines and Their Receptor mRNA Levels by Reverse Transcription (RT)-PCR Array

Gene expression for inflammatory cytokines, chemokines and their receptors was measured using the real-time PCR-based pathway-focused gene profiling “RT2 profiler system” from SuperArray, Inc. (mouse inflammatory cytokines and receptor PCR Array). Procedures were carried out according to the manufacturer's protocol. Briefly, 1 µg of total RNA was used as template for the reverse transcription of cDNA. The cDNA was diluted and added to a master mix containing the fluorescent SYBR Green dye. Aliquots from this mix were added to a 96-well plate: each well contained previously dispensed gene-specific primer sets. The plates were then placed in the Chromo4 (Bio-Rad), and real-time PCR analysis was performed. Each plate contained a panel of housekeeping gene primers for normalizing PCR array data, and estimating the linear dynamic range of the assay. Furthermore, “no reverse transcription” and “no template” samples were included as negative controls for each reaction. Raw data were acquired and processed with the Chromo4 (Bio-Rad) to calculate the threshold cycle (Ct) value and relative gene expression values, subsequently determined according to the standard ΔΔCt method.

### Analyses of Gene mRNA Levels by RT-PCR

Total RNA (2 µg) was reverse transcribed using oligo(dT) primers and Moloney murine leukemia virus reverse transcriptase, according to the manufacturer's instructions (Eurogentec), in a total volume of 20 µl. Synthesized cDNA (2 µl) diluted 1/100 was then amplified by PCR in the Chromo4 (Bio-rad). Hypoxanthine phosphoribosyltransferase mRNA levels were used to normalize RNA quantification. We used various primers for mRNA quantification: the previously described HPRT, IFNγ, IL12, CCL2 and CCL7 primers [14], [17], TLR9 (forward: CCGCCCAGTTTGTCAGAGG; reverse: GGGGTACAGACTTCAGGAACAG), CXCL10 (forward: CACGTGTTGAGATCATTGCCA; reverse: GCGTGGCTTCACTCCAGTTA), CXCL1 (forward: CGCTCGCTTCTC TGTGCAGC; reverse: GTGGCTATGACTTCGGTTTGG), FoxP3 (forward: ATCTCCTGGATGAGAAAGGCAAGG; reverse: TGTTGTGGAAGAACTCTGGGAAGG), and IL10 (forward: GGTTGCCAAGCCTTATCGGA; reverse: CTGCTCCACTGCCTTGCTCTTATT).

### Statistics

Non-parametric analyses were performed with the Mann-Whitney tests.

## Results

### Th1-Type Cytokine Response after CpG-ODN Treatment

**Figure 1:**
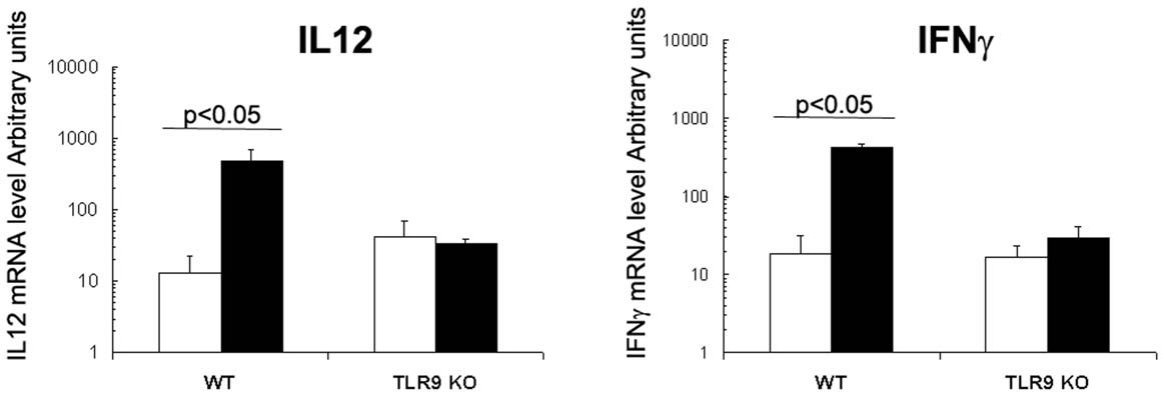
IL12p40 and IFNγ responses after CpG-ODN treatment in the intestine. CpG-ODN was administered orally in 8-day-old WT and TLR9 KO neonate mice at a dose of 20 µg/g (black boxes). Control mice received PBS orally (white boxes). N = 5 neonates from two different litter for each group. Six hours later, ilea were removed for RNA extraction. RT-PCRs were performed for amplification of IL12p40 (A) and IFNγ (B) mRNA. These data are representative of two experiments. Data were analyzed by the non-parametric Mann-Whitney test.

CpG-ODN is known to be a strong Th1 inducer; thus, the capacity of orally administered CpG-ODN to increase IL12p40 and IFNγ mRNA levels in the intestine of 8-day old newborn mice was evaluated by real-time RT-PCR. CpG-ODN given orally significantly increased IFNγ and IL12p40 mRNA levels in comparison with sham-treated litters: mRNA levels were 23- and 37-fold greater, respectively, 6 h after administration (Fig. 1A and B). These data are in agreement with our previous results obtained with 1–2 day-old orally-treated neonates [14]. This response was strictly TLR9-dependent, as this overexpression was absent in the mucosa of TLR9-deficient newborns treated with oral CpG-ODN (Fig. 1A and B).

### Recruitment of Inflammatory Cells into the Intestinal Mucosa and Draining Lymph Nodes of Neonates after Oral Administration of CpG-ODN

**Figure 2:**
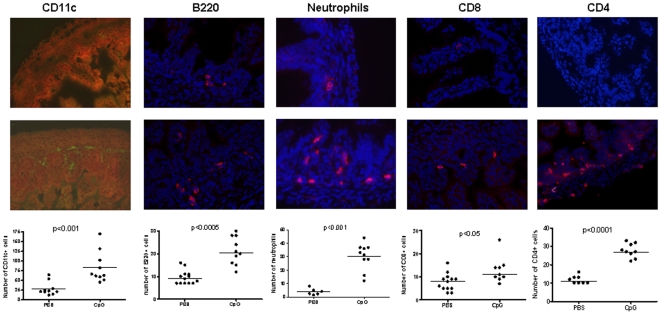
Recruitment of inflammatory cells in the intestine of neonates after CpG-ODN treatment. Eight-day-old neonatal mice received 20 µg/g of CpG-ODN orally. Twenty-four hours after treatment, ilea were removed to analyze the recruitment of inflammatory cells by immunohistology. Six to ten neonates from different litters (5 sections for each animal) per group were analyzed. Data were analyzed by non-parametric Mann-Whitney test.

**Figure 3:**
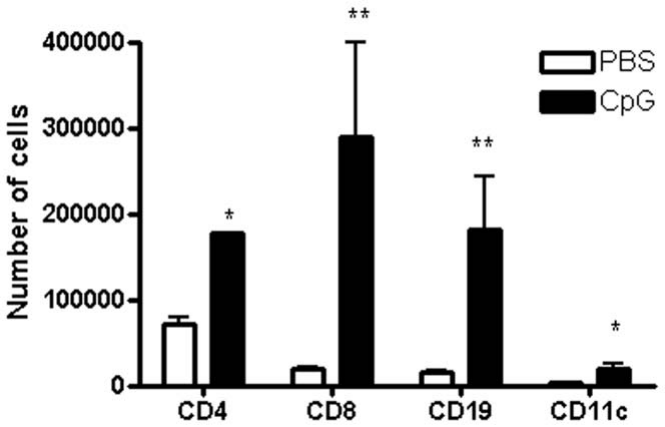
Recruitment of inflammatory cells in the MLN after CpG-ODN treatment. Eight-day-old neonatal mice received 20 µg/g of CpG-ODN by the oral route. Twenty-four hours later, MLN from 10 neonates were removed and pooled for FACS analysis. Four pools in each group were analyzed. These data were from two representative experiments. Mann-Whitney non parametric test ** p<0.005, * p<0.05.

The intestinal mucosa in newborns is significantly less colonized by immune cells than that in adults. We characterized the capacity of orally given CpG-ODN to induce immune cell recruitment in the intestinal mucosa of neonates. Eight-day-old mice received a single oral dose of 20 µg/g of CpG-ODN, and 24 h later a significant recruitment of CD11c+ dendritic cells, B220+ B cells, CD4+ and CD8+ cells and neutrophils was observed in the intestinal mucosa (Fig. 2). Thus, CpG-ODN administered orally induced a rapid recruitment of a broad range of immune cells to the newborn intestinal mucosa. To investigate whether orally given CpG-ODN also triggered cell trafficking into the draining lymph nodes, mesenteric lymph nodes (MLN) were removed from neonates 24 h after treatment with CpG-ODN and the cell composition phenotype was determined by flow cytometry. We observed greater numbers of CD4+ (x3), CD8+ (x5.5), CD19+ (x3) and CD11c+ (x8) cells in neonates after a single dose of CpG-ODN given orally than in those treated with PBS (Fig. 3).

### Chemokine and Cytokine Responses in Mice after Treatment with CpG

**Figure 4:**
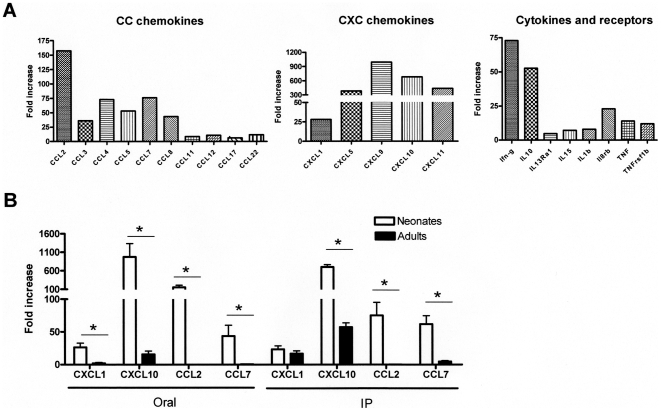
Mucosal immune responses after CpG-ODN treatment in the intestine. CpG-ODN was administered by the oral route in 8-day-old neonates (n = 5 for each group). Six hours later, ilea were removed for RNA extraction. (A) We used an RT-PCR microarray to analyze a pool of RNA for each group. CC, CXC chemokines and cytokines/receptor data for values with greater than 3-fold-increases are represented. (B) Neonates (n = 5) and adults (n = 5) received 20 µg/g CpG-ODN orally and 10 µg/g intraperitoneally. The mice were killed 6 hours later and the ilea were removed. RNAs were extracted and RT-PCR analyses were performed for CCL2, CCL7, CXCL1 and CXCL10 expression. These data are representative of two independent experiments. Data were submitted to the non-parametric Mann-Whitney test (*, p<0.05).

To better characterize the inflammatory response induced in neonates by CpG-ODN treatment, chemokines, small proteins principally involved in inflammation regulation and leukocyte trafficking, were measured by an RT-PCR array. This assay allows the simultaneous analysis of mRNA levels for 84 genes encoding inflammatory cytokines, chemokines and their receptors. Immune responses in the ileum of neonates were analyzed 6 h after oral CpG-ODN treatment. A very high overexpression of three CXC-nonELR chemokines (CXCL9, 10, 11) was observed (680-, 989-, and 436-fold increases, respectively) (Fig. 4A). CXCL9, 10 and 11 exert their functional effects through interactions with a specific high-affinity G-protein–coupled receptor, CXCR3, which is expressed by activated memory T-cells, predominantly of a Th1 phenotype, and which has been detected in T-cells isolated from a diverse number of inflammatory tissues [18]. mRNA levels of CXCL5 and CXCL1, known to recruit neutrophils, were also increased (380- and 28-fold greater, respectively). Other ELR+-CXC chemokine mRNA levels, such as those for CXCL4, 12, 13 and 15, did not vary after CpG-ODN treatment. Among the CC chemokines analyzed, increases of CCL2 (157-fold), CCL4 and CCL7 (73- and 76-fold, respectively), CCL3 and CCL5 (36- and 53-fold, respectively) and to a lesser extent those of CCL11, CCL12, CCL17 and CCL22 (6- to 11-fold increases) were observed. Several interleukins were also been analyzed with this method, and the pro-inflammatory cytokine IL1β (8-fold greater), the regulatory cytokine IL10 (52-fold greater) and IL15 (7-fold greater) were up-regulated after CpG-ODN treatment. TNFα mRNA levels were also moderately greater than in individuals without treatment (14-fold) (Fig. 4A). Finally, our analysis of cytokine or chemokine receptor mRNA production did not reveal any significant change, suggesting that 6 h of stimulation was not sufficient to either upregulate receptor mRNA levels in the few immune cells present in the mucosa, or more probably was insufficient at inducing a significant recruitment of immune cells bearing receptors in the mucosa (data not shown).

### Comparison of Mucosal Chemokine Responses between Adult and Neonatal Mice after Oral or Intraperitoneal Administration of CpG-ODN

We selected two CC and two CXC chemokines for which over expression in the mucosa of neonates treated orally with CpG-ODN was high: CCL2, CCL7, CXCL1 and CXCL10 were chosen to compare the efficiency of orally administered CpG-ODN to stimulate intestinal immune responses in neonates and in adult mice. A real-time RT-PCR analysis revealed that the amounts of CCL2 and CCL7 mRNA were greater in neonates after oral CpG administration (154-fold and 44-fold greater, respectively) than in adults (Fig. 4B). We also observed higher CXCL10 and CXCL1 mRNA levels in neonatal intestinal mucosa (neonates: 975-fold and 26-fold greater, respectively; adult: 15-fold and 3-fold greater, respectively). We then wondered if this effect was specific to the oral route. After IP administration, an increased expression of CXCL10 (57-fold), CXCL1 (16-fold) and CCL7 (5-fold) in the mucosa of adult mice was observed, but these upregulations were weaker than those observed in neonates (700-, 23-, 75- and 62-fold greater for CXCL10, CXCL1, CCL2 and CCL7 respectively). Taken together, these results showed that neonates responded more efficiently to CpG-ODN than adult mice, and that administering CpG-ODN orally is particularly efficient in neonates.

### Response of Freshly Purified Intestinal Epithelial Cells (IEC) to In Vitro Stimulation with CpG-ODN

**Figure 5:**
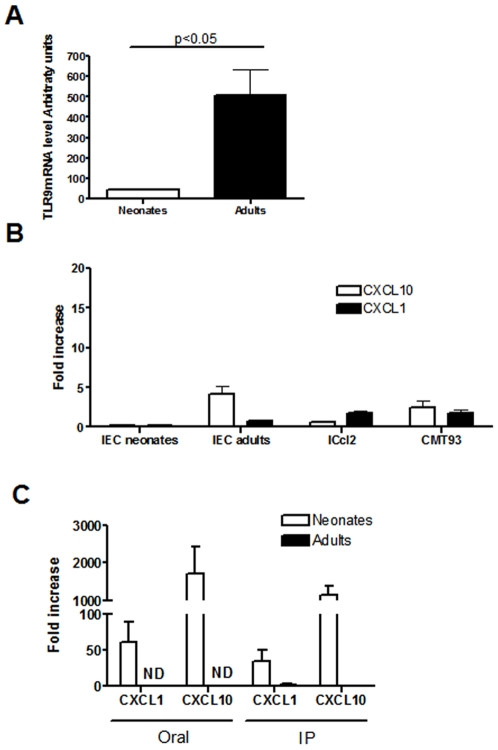
Chemokine responses of intestinal epithelial cells after CpG-ODN stimulation. (A) Intestinal epithelial cells (IEC) were purified from neonatal (n = 5) or adult mice (n = 5). RNA was extracted from the purified cells for TLR9 mRNA RT-PCR analysis. (B) Two murine intestinal epithelial cell lines (ICcl2 and CMT93) and purified IECs from adults and neonates were stimulated in vitro with 5 µg/ml of CpG-ODN for 2 hours, and RNAs were extracted for CXCL1 and CXCL10 measurement. (C) Neonatal (n = 30 for each group) and adult mice (n = 3 for each group) received CpG-ODN by the oral and IP route. They were killed 6 hours later, and the IECs were purified. RNAs were extracted and RT-PCR analyses were performed for CXCL1 and CXCL10 expression. Data were submitted to the non-parametric Mann-Whitney test. (*, p<0.05). ND, Not Done.

Intestinal epithelial cells are the first cells associated with the digestive tract that might respond to oral adjuvant stimulation. We compared mRNA levels of the CpG-ODN receptor, TLR9, in IECs freshly purified from adult and neonates, to study how neonate intestinal epithelial cells (IEC) contribute to the stimulation of efficient innate immune responses triggered by CpG-ODN. IECs from adults had about 10 times more mRNA than neonates (Fig. 5A). When freshly purified IECs were stimulated *in vitro* with CpG-ODN, neonatal IECs did not respond to stimulation; CXCL10 mRNA levels in adults were about 5-fold-greater than without CpG-ODN (Fig. 5B). This low overexpression was consistant with that observed within mouse IEC lines ICcl2 and CMT93 stimulated with CpG-ODN (Fig. 5B).

### Responses of Purified IECs from Adults and Neonates after In Vivo Administration of CpG-ODN

Both the cellular environment and polarization of IECs may influence responses to CpG-ODN. Therefore, in vivo CpG-ODN stimulation was compared for drug administration by the oral and IP route. IECs purified from neonates that received CpG-ODN by oral or IP route displayed greater amounts of CXCL10 mRNA (1720- and 1144-fold greater, respectively) and CXCL1 mRNA (61- and 35-fold-greater, respectively), whereas those from adults that received CpG-ODN by the IP route did not (Fig. 5C). No expression of CCL2 and CCL7 chemokines has been detected in freshly purified IEC preparations from adult and wild-type neonates (data not shown). This suggests that IECs are key contributors to CXCL1 and CXCL10 responses, but not to CCL2 and CCL7 responses measured in the mucosa of neonates.

### Role of IL10 in Cytokine and Chemokine Responses after CpG-ODN Administration

**Figure 6:**
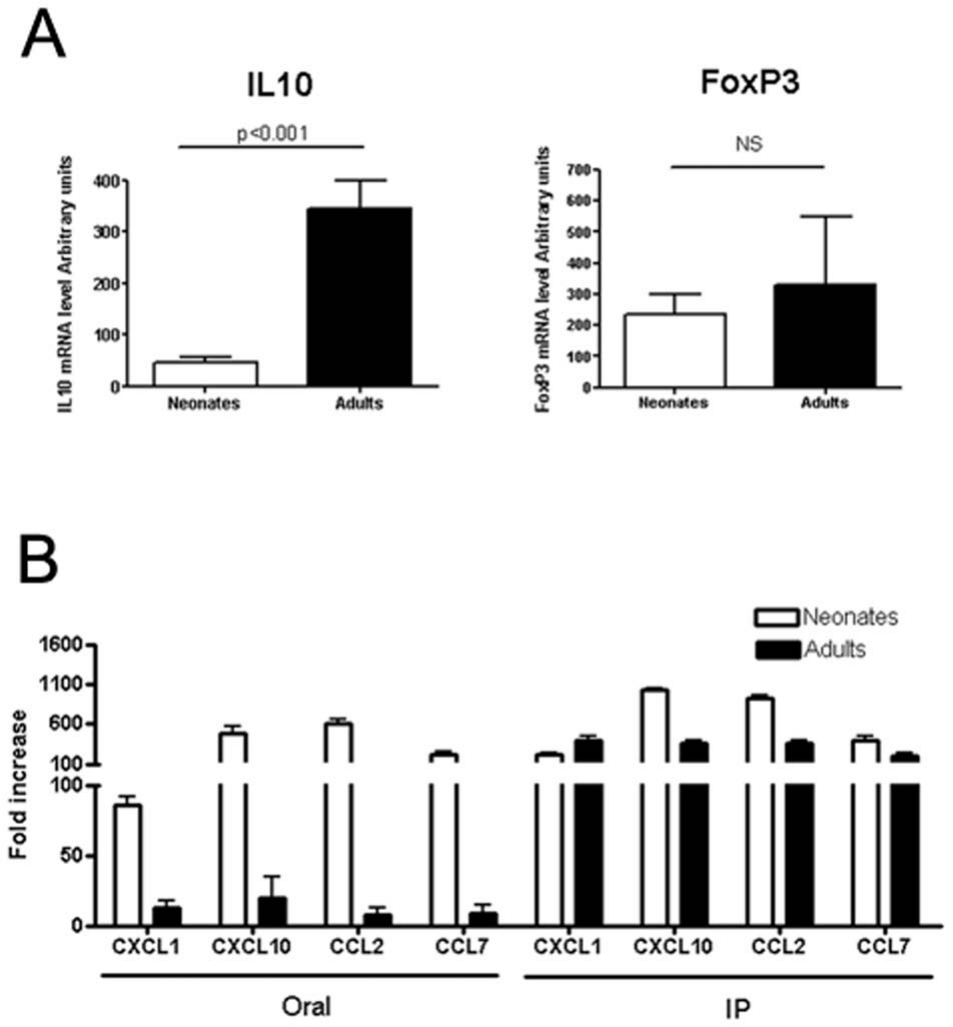
Role of IL10 in the chemokine response induced by CpG-ODN stimulation. (A) Ilea of 8-day old neonatal (n = 8) and of adult (n = 6) mice were removed for RNA extraction. IL10 and FoxP3 mRNA levels were measured by real-time RT-PCR. Data were analyzed by the non-parametric Mann-Whitney test. (B) Neonates (n = 3) and adults (n = 3) IL10-deficient mice received PBS or 20 µg/g CpG-ODN orally and 10 µg/g intraperitoneally. They were killed 6 hours later and the ilea were removed. Total RNAs were extracted and RT-PCR analyses were performed for CXCL1, CXCL10, CCL2 and CCL7.

IL10 is a regulatory cytokine produced by DCreg, Treg or Breg cells. In the intestine of adult mice, constitutive IL10 impairs dendritic cell TLR stimulation from lamina propria [19]. Constitutive IL10 expression was higher (7-fold-higher) in the intestine of adult mice than in neonatal intestines (Fig. 6A). Foxp3 expression is a hallmark of T regulatory cells that might produce IL-10. In the intestine of adults the number of Foxp3+T cells was not increased as suggested by the similar level of Foxp3 mRNA expression within adults and neonates (Fig. 6A). We next wondered if this difference in the constitutive IL10 mRNA level between adults and neonates could explain the difference in chemokine responses after CpG administration. To address this question, IL10KO adult and neonatal mice received CpG-ODN by the oral or IP route, and the intestinal chemokine response was measured. As for wild-type mice, the administration of CpG-ODN by oral route to IL10KO mice is much more efficient in neonates than in adult to stimulate an intestinal chemokine response (Fig. 6B). In contrast, in the absence of IL10, CpG-ODN administration by IP route in adult mice induced an over-expression of CXCL1 (400-fold greater), CXL10 (356-fold greater), CCL2 (357-fold greater) and CCL7 (205-fold greater) (Fig. 6B). These chemokine over-expressions were about 10- to 20-fold higher and even about 300-fold for CCL2 than those observed in adult IL10-sufficient mice (Fig. 6B). These results clearly demonstrate than IL10 can negatively control CpG-ODN-induced chemokine responses. It also suggests that higher levels of IL10 at homeostasis in the adult intestine may contribute to the lower chemokine responses after CpG treatment than observed in neonatal mice.

## Discussion

Neonates are highly sensitive to intestinal and respiratory infection due to immune system immaturity [20]. Functional and quantitative deficiencies, involving both non-specific innate defenses and specific cellular and humoral defenses, have already been described in newborns [2]. However, little is known about neonatal gut mucosal immunology. The intestinal compartment in neonates is almost devoid of immune cells and this could contribute to the high sensitivity observed in response to intestinal pathogens [17], [21], [22]. It is therefore of a particular interest to develop strategies for the recruitment of immune cells in neonates and for stimulating their intestinal immune system to improve their resistance to infection. In this study, CpG-ODN, a potent adjuvant and immunostimulant, was used to investigate its effect on the intestinal immune response of neonates. Neonates displayed an increased intestinal chemokine response after an oral administration of CpG-ODN, which was associated with a large recruitment of inflammatory cells. Surprisingly, this chemokine response was much stronger than in adults. We therefore tried to gain further insight into mechanisms in the intestinal immune system of neonates responsible for this higher chemokine responsiveness to orally administered CpG-ODN.

The immune effects of CpG-ODN are well described when given by parenteral route, but little is known of its effects when given orally. Neonatal mice receiving an oral dose of CpG-ODN exhibited an increased intestinal response in chemokines belonging to both CC and CXC chemokine families. The strong CXCL5 response and the CCL2, 3, 4 and 5 responses may have induced, respectively, the recruitment of neutrophils and dendritic cells observed 24 h after CpG-ODN administration. The upregulation of the three nonELR-CXC chemokines CXCL9, 10 and 11 can be linked to the significant recruitment of T cells. A production of chemokines such as CCL2, 4, 5 and CXCL10 after an oral administration of CpG was previously observed in the intestine of adult mice [13], [23]. Chemokine responses in the intestine of neonates receiving CpG-ODN orally were much stronger than those in adults. As IECs express TLR9 on their basal and apical side [24], and are the first cells in contact with orally administered CpG, these cells might contribute to these differences between neonates and adults. Although basolateral TLR9 signals IκBα degradation and activation of the NF-κB pathway, apical TLR9 stimulation invokes a unique response in which ubiquitinated IκB accumulates in the cytoplasm preventing NF-κB activation [24]. This polarized response to CpG-ODN may explain the higher chemokine response that we observed in the intestinal mucosa of neonates compared with the response seen in adults. Closure of the intestinal epithelium in neonates occurs after about 3 weeks [25], and CpG-ODN given orally may have access to the basolateral side of neonatal IECs, activating the NF-κB pathway and producing chemokines such as CXCL1 and CXCL10. However, direct in vitro stimulation of freshly purified IECs with CpG-ODN, thus providing access to both sides of IECs, presented CXCL10 mRNA levels in adults that were only slightly greater than without CpG-ODN; moreover, no changes were observed in neonatal IECs. This overexpression of CXCL10 was however significantly weaker than that observed in neonatal IECs purified after in vivo stimulation, suggesting that intestinal epithelial cells are poorly responsive to direct CpG-ODN stimulation whatever the age of the animal. There is increased evidence showing that IECs are generally tolerogenic to direct TLR stimulation, avoiding uncontrolled inflammation that is detrimental to the host. Several mechanisms relating to TLR stimulation in IECs have been previously described, such as the induction of negative regulators that include IRAK-M, TOLLIP, and SIGIRR [26]. However, there are few studies on the responses of neonatal IECs to TLR stimulation. Similar to our findings with CpG-ODN, Lotz *et al.* found that TLR4 stimulation with LPS increases KC and MIP2 chemokine production by IECs in fetuses and neonates, but that this response was absent in adults. [22]. Our results suggest that IECs are also tolerogenic to TLR9 stimulation, as early as the first week after birth; This may occur to facilitate microbial colonization in neonates and an intestinal host–microbe homeostasis in adults.

IECs are poorly responsive to direct *in vitro* TLR9 stimulation. However, our data with IECs purified after in vivo stimulation with CpG show that they are major contributors to the CXCL1 and CXCL10 chemokine response measured in the intestinal mucosa of neonates, and most probably play a role in the neutrophil recruitment observed. Thus, our findings suggest that other cells of the lamina propria produce CCL chemokines. These cells probably contribute to the production of soluble factors that can indirectly stimulate IECs for CXC chemokine production. Differences in permeability between the intestinal epithelium of adult and neonatal mice could explain why, in adults, the immune cells of the lamina propria were less accessible to orally administered CpG-ODN, leading to a weaker intestinal chemokine response both in wild-type and IL10 KO mice. However, this cannot explain why wild-type adult mice presented weaker CCL2, CCL7 and CXCL10 intestinal responses after intraperitoneal administration of CpG-ODN than neonatal mice. Indeed, we could have expected a stronger response to CpG-ODN given by the IP route in adult mice, due to the larger number of immune cells bearing TLR9 in the lamina propria, than in neonates. These results suggest that adult immune cells in the lamina propria are less responsive to CpG-ODN than neonatal ones. These results are consistent with other results on neonatal immune cells. By using a model of kid neonates, we recently reported that neonatal cells from mesenteric lymph nodes display a stronger TH1-type cytokine response to TLR ligands than adults [27]. As intestinal flora may affect TLR-ligand responsiveness, we performed an experiment with germ free neonate and adult mice. Similar levels of chemokine expression were observed following IP administration of CpG-ODN demonstrating that the intestinal flora as no role in the difference of response observed between neonates and adults (data not shown). We then addressed whether neonatal mouse intestines have fewer regulatory components than adults. Despite FoxP3 mRNA expression, the hallmark of regulatory T cells with similar expression levels in the mucosa of adults and neonates, we observed IL10 mRNA levels in adult mouse mucosae that were seven-fold greater than in neonates. This suggests that this regulatory cytokine, produced by either B or dendritic cells, could contribute to the reduced response to TLR9 stimulation in adult mice. Adult IL10-deficient mice intraperitoneally stimulated with CpG-ODN exhibited a stronger chemokine response than wild-type adult mice, and display similar chemokine levels as IL10KO neonatal mice that received CpG-ODN intraperitoneally. Therefore IL-10 intestinal levels affect chemokine responsiveness to TLR9 stimulation. Moreover, as wild-type neonates express low levels of IL-10 in their intestine, the chemokine over-expressions observed in IL-10 KO neonatal mice after CpG-ODN administration were similar to the ones of wild-type mice. Monteleone et al. have recently shown that dendritic cells from adult mouse lamina propria are less responsive to TLR stimulations due to a high constitutive production of IL10. This is despite higher levels of TLR2, 3, 4 and 9 proteins than those found in spleen and MLN DCs [19]. In the sheep model, Peyer's patch cells spontaneously secrete high levels of IL-10 and exhibit poor cytokine responses (IFN-α, IFN-γ and IL-12) following stimulation with CpG-ODN compared with peripheral blood mononuclear cells and lymph node cells [28]. Our findings are in accordance with these observations, and clearly show that IL10 is involved in the negative control of the intestinal immune response to TLR9-stimulation in adult mice.

To summarize, CpG-ODN administered by oral route is very efficient in neonates to induce a CCL and CXC chemokine response and the recruitment of a panel of inflammatory cells in the intestine. IECs respond indirectly to CpG-ODN and are major contributors in the CXC chemokine response, whereas CC chemokines seem to be produced by other cells of the lamina propria, cells that are probably also the source of Th1-type cytokines. Taken together, our findings suggest that the efficacy of the oral route in neonates compared with adults is due to two additional factors: the higher permeability of the intestinal barrier before the closure of the epithelium, and an intestinal environment with lower levels of IL10. Inducing this local immune response could provide a great opportunity for controlling intestinal intracellular pathogens in neonates, as we have previously reported for cryptosporidiosis [14].
